# Pathological Importance of the Endothelin-1/ET_B_ Receptor System on Vascular Diseases

**DOI:** 10.1155/2012/731970

**Published:** 2012-07-30

**Authors:** Kento Kitada, Mamoru Ohkita, Yasuo Matsumura

**Affiliations:** ^1^Laboratory of Pathological and Molecular Pharmacology, Osaka University of Pharmaceutical Sciences, 4-20-1 Nasahara, Takatsuki, Osaka 569-1094, Japan; ^2^Department of Pharmacology, Kagawa University, Kita-gun, Kagawa 760-0016, Japan

## Abstract

Activation of the endothelin (ET)-1/ET receptor system is involved in the development of vascular diseases such as atherosclerosis, vascular hypertrophy, and restenosis. Some issues still remain unresolved including whether ET receptor antagonists are expected to become the new therapeutic tools for the treatment of vascular diseases. One of the unresolved critical points is the functional role of ET receptor subtypes on each vascular disease, in particular the pathophysiological roles of the ET_B_ receptor. We recently demonstrated that selective inhibition of the ET_B_ receptor system showed harmful effects in the development of neointimal formation after vascular injury. However, there was no apparent difference in the therapeutic effects between a nonselective ET_A_/ET_B_ receptor antagonist and selective ET_A_ receptor antagonist. These findings indicate that antagonism of the ET_A_ receptor system is essential for suppressing vascular remodeling, irrespective of the presence of ET_B_-receptor-mediated actions, although the selective ET_B_ receptor antagonist worsens vascular remodeling. In addition, we found that ET receptor systems contribute to sex differences in the severity of vascular disease, thereby suggesting that the efficacy of ET receptor antagonists for vascular diseases may differ between sexes. In this paper, we outline the roles of the ET-1/ET_B_ receptor system on vascular diseases and its sex differences.

## 1. Introduction

Endothelin (ET)-1 was discovered as a potent and long-lasting vasoconstrictive peptide derived from endothelial cells [1]. ET-1 induces various actions to vessels such as vasoconstriction, vasodilation, and vascular cell proliferation via ET_A_ and ET_B_ receptors [2–4]. From previous clinical and basic studies, it has been reported that the ET-1/ET receptor system is one of the critical factors for the development of hypertension and cardiovascular diseases [2–4]. Pathological activation of the ET-1/ET receptor system could play important roles in the development of hypertension, pulmonary hypertension, vascular remodeling (arteriosclerosis and restenosis), myocardial infarction, heart failure, and renal failure [2–4]. A number of studies have been trying to develop an ET receptor antagonist or ET-1 synthesis inhibitor as a new therapeutic tool for hypertension and cardiovascular diseases. So far, an ET_A_/ET_B_ dual receptor antagonist and selective ET_A_ receptor antagonist have been used as therapeutic agents of pulmonary hypertension. Although there is increasing evidence regarding the cardioprotective and vasoprotective effects of ET receptor antagonists, several issues still remain to be resolved, that is, the pathophysiological roles of ET receptor subtypes (especially the ET_B_ receptor) in each disease have not been fully elucidated yet. It is one of the critical points for clinical application as to which type of ET receptor antagonist is a better medicine for the treatment of each disease.

## 2. Vascular ET-1/ET Receptor System in ****a Physiological State

Vascular endothelial cells mainly produce and secrete ET-1 in vessels. Briefly, big ET-1 is formed from the precursor preproET-1 and mature ET-1 is then produced by endothelin-converting enzyme (ECE). One of the essential actions of ET-1 is a potent and long-lasting vasoconstrictive effect in vascular smooth muscle cells (VSMCs). Thus, ET-1 blockers have attracted attention as an antihypertensive drug. Along with its strong vasoconstrictive action, ET-1 has a cellular proliferative action in VSMCs [5]. ET-1 causes these vascular effects via ET_A_ and ET_B_ receptors. Both ET_A_ and ET_B_ receptors are located on VSMCs and induce vasoconstriction and cell proliferation. ET_B_ receptors are also expressed on endothelial cells as well as VSMCs. Endothelial ET_B_ receptor mediates vasodilative and antiproliferative actions at least partly via NO production in contrast to its function in VSMCs [6]. Thus, ET_B_ receptors have two kinds of actions in the physiological regulation of vasculature. In addition, the ET_B_ receptor is also well known as a clearance receptor of ET-1 from the circulation [7]. In fact, selective ET_B_ receptor antagonist-treated and ET_B_-deficient rats exhibited increases in plasma ET-1 levels [8, 9].

## 3. Vascular ET-1/ET Receptor System in ****a Pathological State

It has been reported that ET-1 contributes to the development of vascular diseases by having a local effect in addition to its systemic hypertensive effects [2–4, 10, 11]. There are various mechanisms underlying ET-1-induced vascular disorders, such as the induction of inflammation and oxidative stress, increases in growth factors (PDGF, FGF) and proliferative factors (EGF), and production of collagen and extracellular matrix [2–4, 10, 11]. One of the key factors regarding vascular diseases is ET-1-mediated VSMCs proliferation. In fact, clinical and basic studies have indicated that proliferation of VSMCs and neointimal formation in response to ET-1 stimulation play a key role in several vascular lesions such as atherosclerosis, restenosis, and arterial hypertrophy by hypertension or diabetes [10, 11]. 

There is basic and clinical evidence that has shown activation of the ET-1/ET receptor system in vascular remodeling sites and development of vascular remodeling. In animal model studies, it was reported that an injured artery after balloon injury exhibited increases in mRNA levels of ET-1, ECE, ET_A_, and ET_B_ receptors [12]. Moreover, continuous ET-1 infusion aggravated neointimal formation after balloon injury [13]. These studies indicate that activation of the ET-1/ET receptor system is involved in the development of vascular remodeling after vascular injury. Actually, a selective ET_A_ receptor and ET_A_/ET_B_ dual receptor antagonist show vasoprotective effects via inhibition of neointimal formation after balloon injury [9, 13–15]. In a clinical study, it was reported that ET-1 levels in the coronary circulation were increased after percutaneous transluminal coronary angioplasty (PTCA) [16]. Furthermore, Shirai et al. [17] reported that neointimal VSMCs after PTCA exhibited enhanced expressions of ECE, ET-1, and ET receptors. This basic and clinical evidence suggests that the ET-1/ET receptor system contributes to the pathogenesis of neointimal formation after vascular injury. Thus, ET receptor antagonists may be useful for the prevention of restenosis after PTCA.

It has been reported that pathological activation of the ET-1/ET receptor system is involved in not only vascular remodeling but also arteriosclerosis. In the arteriosclerotic site of animal models and human patients, ET-1 and its receptor expressions are known to be upregulated [18–20]. Furthermore, ET receptor antagonists could suppress the development of arteriosclerosis in animal models such as LDL receptor- and ApoE-knockout mice [21, 22]. These reports indicated that ET receptor antagonists hold promise for treating arteriosclerosis. Moreover, other papers reported that ET receptor antagonists also suppressed the development of hypertension- and diabetes-induced vascular hypertrophy [23, 24]. These findings suggest that the ET-1/ET receptor system plays an important role in various vascular diseases and that an ET receptor antagonist has vasoprotective effects in the development of various vascular diseases. 

**Figure 1:**
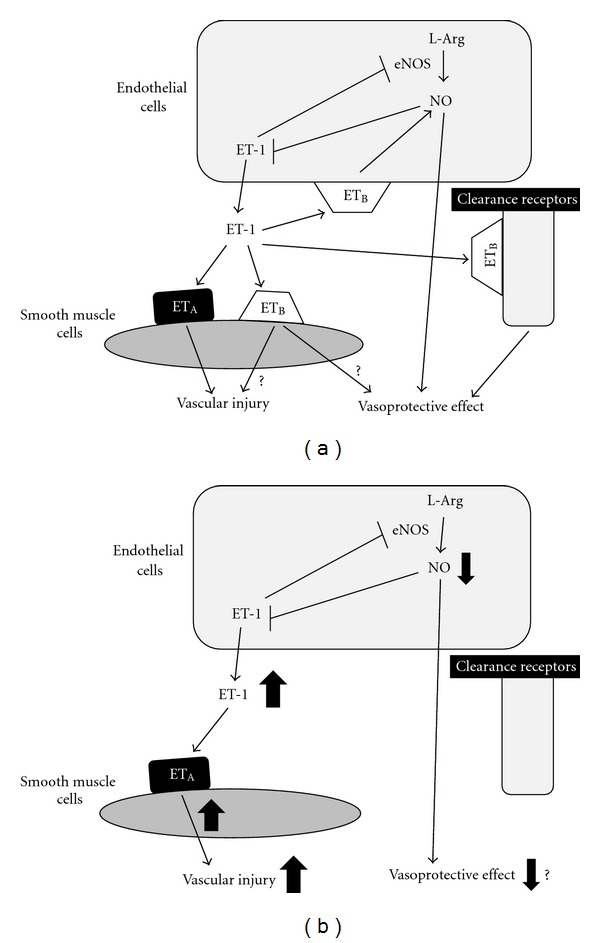
Roles of the ET-1/ET receptor system on vascular injury. (a) The vascular effects of ET-1 are mediated by ET_A_ and ET_B_ receptors. ET_A_-mediated ET-1 action has been considered to cause vascular injury. ET_B_ receptors are expressed not only on vascular smooth muscle cells, but also on endothelial cells. In addition, the ET_B_ receptor is a clearance receptor of ET-1 from the circulation. However, the roles of the ET_B_ receptor on vascular injury are still controversial. Recently, it was reported that nonendothelial ET_B_-receptor induced vasoprotective effects, whereas another previous study demonstrated that ET_B_-receptor-mediated ET-1 action exhibited vasoprotective effects via NO production [25, 26]. (b) Inhibition of the ET_B_ receptor system leads to an aggravation of vascular injury. Increased circulating ET-1 levels because of clearance receptor inhibition and the augmentation of ET_A_-mediated actions are mainly responsible for aggravated vascular injury in the ET_B_ receptor-inhibited condition [9]. ET, endothelin; NO, nitric oxide; eNOS; endothelial nitric oxide synthesis; L-Arg. L-Arginine.

For the treatment of vascular diseases with ET antagonists, which type of ET antagonist is the better choice? There is general agreement that the ET-1/ET_A_ receptor system plays an important role in the development of vascular disease because ET_A_ receptor-mediated ET-1 actions induce VSMCs proliferation and both selective ET_A_ receptor and ET_A_/ET_B_ dual receptor antagonists showed vasoprotective effects. However, the pathological role of the ET-1/ET_B_ receptor system on vascular diseases has not been fully elucidated because of the opposite effects of endothelial and VSMC ET_B_ receptor-mediated actions (Figure 1(a)). Therefore, it is still difficult to answer which type of ET antagonist is more effective for the treatment of vascular disease.

## 4. Pathophysiological Roles of the ET_B_ Receptor in Vascular Diseases

To identify the functional influences of the ET_B_ receptor on vascular diseases, we investigated the effects of ET_B_ receptor blockade at pharmacological or genetic levels on the development of neointimal formation after balloon injury. We clearly demonstrated that inhibition of the ET_B_ receptor system, using a selective ET_B_ receptor antagonist or ET_B_-deficient rat, aggravated neointimal formation after balloon injury [9]. These results led us to propose that the ET-1/ET_B_ receptor system is protective in the pathogenesis of neointimal formation after balloon injury and that a selective ET_A_ receptor antagonist may be more effective than a nonselective ET_A_/ET_B_ receptor antagonist for the prevention of neointimal formation. Interestingly, an ET_A_/ET_B_ dual receptor antagonist as well as a selective ET_A_ receptor antagonist markedly suppressed the development of neointimal formation, and the efficacy of treatment was comparable between the above two types of antagonists [9]. Furthermore, aggravated neointimal formation observed in ET_B_-deficient rats was dramatically improved by the inhibition of the ET_A_ receptor [9], suggesting that aggravated neointimal formation after balloon injury in the ET_B_ receptor-inhibited condition can be prevented by pharmacological blockade of ET_A_ receptors. In addition, antagonism of the ET_B_ receptor itself does not seem to impair the positive effects of concomitant ET_A_ receptor antagonism. These findings may lead us to propose an important conclusion where chronic inhibition of ET_B_ receptors leads to an overstimulation and/or upregulation of the ET_A_ receptor system; therefore, it seems likely that an augmentation in ET_A_ receptor-mediated ET-1 actions is an essential factor for the enhancement of neointimal formation observed in ET_B_ receptor antagonist-treated and ET_B_-deficient rats rather than blockade of the ET_B_ receptor-mediated vasoprotective effect (Figure 1(b)). In other words, antagonism of the ET_A_ receptor is essential for the inhibition of neointimal formation after balloon injury, irrespective of the presence of ET_B_ receptor-mediated actions. This hypothesis could explain why both selective ET_A_ receptor and nonselective ET_A_/ET_B_ dual receptor antagonists are equally effective in suppressing neointimal formation [9, 13–15].

Other disease models such as hypertension and pulmonary hypertension, as well as the balloon injury model, showed overactivation of the ET_A_ receptor system in the ET_B_ receptor-inhibited condition, but the detailed mechanism has not been fully elucidated yet [9, 24, 27, 28]. One possible factor is an increase in plasma ET-1 concentrations in the ET_B_ receptor-inhibited condition because ET_B_ receptors work as a clearance receptor of ET-1 from the circulation (Figure 1(b)) [7–9]. Further research is required to explain why ET_A_ receptor action is augmented in the ET_B_-inhibited condition.

As described above, we demonstrated that selective inhibition of the ET_B_ receptor induces a harmful effect in the balloon injury model. Murakoshi et al. [25] also reported that vascular remodeling caused by a vascular ligation model was markedly enhanced in ET_B_-receptor-knockout mice or selective ET_B_ receptor antagonist-treated mice, whereas selective ET_A_ receptor blockade suppressed this vascular remodeling in mice. Furthermore, Sachidanandam et al. [29, 30] found that resistance artery remodeling in diabetic rats was aggravated by a selective ET_B_ receptor antagonist, in contrast to the beneficial effects of a selective ET_A_ receptor antagonist and ET_A_/ET_B_ receptor antagonist. Taken together, selective inhibition of ET_B_ receptors causes harmful effects in several vascular disease models. However, it is unclear whether ET_B_ receptor-mediated vasoprotective actions are directly responsible for aggravated vascular diseases in the ET_B_ receptor-inhibited condition, and whether a selective ET_A_ receptor antagonist is more effective than an ET_A_/ET_B_ receptor antagonist. In the case of a ligation-induced vascular remodeling model, the efficacy of a selective ET_A_ receptor antagonist is more in wild-type mice than in ET_B_-knockout mice [25], suggesting that the ET_B_ receptor system itself exerts an aggressive vasoprotective effect on vascular disease, in contrast to our findings obtained using a balloon injury model [9]. Why are the roles of the ET_B_ receptor are different between ligation- and balloon injury-induced vascular injury models? One of the possible answers is differences between vascular injury models. The balloon injury model causes endothelial injury, therefore, there are no endothelial cells in the injured artery site after balloon injury. Meanwhile, endothelial ET_B_ receptor stimulation in a vascular ligation model can mediate NO production and anti-proliferative action because vascular endothelial cells exist in this model. Actually, it was reported that tissue NOx levels are lower in the injured artery site of ET_B_-knockout mice than that of wild-type mice [25]. Therefore, vascular remodeling models that have endothelial cells may indicate active vasoprotective effects of the ET_B_ receptor in contrast to the endothelium-injury model. 

However, recent evidence does not support the above view. Kirkby et al. [26] demonstrated that nonendothelial cell ET_B_ receptors could limit the development of neointimal formation after wire injury using endothelial cell-specific ET_B_ knockout mice. That is, selective deletion of ET_B_ receptors from the endothelium had no effect on neointimal formation after vascular injury whereas a selective ET_B_ receptor antagonist aggravated neointimal formation after vascular injury in mice [26]. Furthermore, they showed that a selective ET_B_ receptor antagonist reversed the vasoprotective effects of a selective ET_A_ receptor antagonist in the same model [26]. These findings indicate that nonendothelial ET_B_ receptors have aggressive vasoprotective effects and that selective ET_A_ receptor antagonists are preferable to ET_A_/ET_B_ receptor antagonists for the treatment of vascular injury. Therefore, their observations regarding the vasoprotective effects of ET_B_ receptors are completely different from our results. At present, we cannot explain why these differences in ET_B_ receptor function occurred in each model. Further efforts to elucidate the roles of both endothelial and nonendothelial ET_B_ receptors are needed in future studies.

There are still some important questions regarding ET_B_ receptors in vascular diseases. However, the same findings from previous studies are that selective inhibition of the ET_B_ receptor system aggravates vascular remodeling in contrast to the beneficial effects of ET_A_ receptor blockade. Therefore, a selective ET_A_ receptor antagonist may be superior to an ET_A_/ET_B_ dual receptor antagonist in vascular diseases under existing conditions.

However, it still remains unclear in human patients in the priority of the treatment by an ET_A_/ET_B_ dual receptor antagonist or a selective ET_A_ receptor antagonist in the cardiovascular diseases. This will need the randomized, double-blinded clinical trial in the patients with cardiovascular diseases.

## 5. ET-1/ET Receptor System and Sex Differences in Vascular Diseases

Epidemiology-based clinical investigations have demonstrated that the incidence of cardiovascular disease is lower in premenopausal women than that of men and postmenopausal women [31, 32]. Although the detailed mechanisms to explain this cardiovascular disease-related sex difference have not been fully elucidated, the vasoprotective effects of estrogen are contributive at least partly to this sex difference [33–35]. It is well known that estrogen has pleiotropic vasoprotective effects via several mechanisms such as upregulation of endothelial NO production and downregulation of adhesion molecule activity, smooth muscle proliferation/migration, and superoxide production [36–38].

On the other hand, it has been indicated that ET-1/ET receptor systems contribute to the sex difference of cardiovascular diseases and hypertension [39, 40]. There is some clinical evidence showing the association between ET-1 and sex differences in the cardiovascular system. Plasma ET-1 concentrations are higher in men than women, and older women show high plasma ET-1 levels [41–43]. Interestingly, plasma ET-1 levels are known to change after sex-change operations [44]. Meanwhile, 17*β*-estradiol treatment in postmenopausal women decreases plasma ET-1 levels [41]. Basic studies also indicated that estrogen suppressed ET-1 production and its action [39, 45]. These reports suggest that ET-1 systems are closely related to the mechanisms of sex differences in cardiovascular diseases and seem to contribute to the increase in incidence of cardiovascular events in postmenopausal women.

Moreover, there are some reports regarding the possible involvement of ET receptors in the sex differences in vessel function. In human saphenous veins, men exhibit higher total number of ET-1 receptors as well as a higher ratio of ET_A_ to ET_B_ receptors than those of women [46]. In deoxycorticosterone acetate/salt-induced hypertension rats, vascular mRNA expression of ET_B_ receptors is higher in males than that in females [47, 48]. Moreover, it has been reported that endogenous estrogen or exogenous 17*β*-estradiol treatment regulates ET receptor gene expression in vessels [47, 49]. These clinical and basic studies suggest that changes in ET receptor distribution in the vasculature may play an important role in the mechanism of sex differences of cardiovascular diseases and/or hypertension.

## 6. Roles of the ET_B_ Receptor in the Sex**** Differences of Vascular Diseases

In animal models of vascular lesions such as neointimal formation after vascular injury, male rats developed a more robust neointimal response to vascular injury than females. Furthermore, neointimal formation in females was augmented by ovariectomy and this augmentation was abolished by 17*β*-estradiol replacement [50]. Thus, there are clear sex differences about neointimal formation after vascular injury in animal models. The mechanisms underlying this sex difference have not been fully elucidated, but are considered to be at least partly related to the vasoprotective actions of estrogen [50].

We recently found that the function of ET_B_ receptors is involved in the sex differences for the development of neointimal formation after balloon injury in rats. In that study, the extent of neointimal formation after balloon injury in wild-type rats was much lower in females than in males [8]. In contrast, in ET_B_-deficient rats, the incidence of neointimal formation after balloon injury was markedly increased to the same extent in males and females [8]. Furthermore, treatment with a selective ET_B_ receptor antagonist also abolished the sex differences of balloon injury-induced neointimal formation in rats [8]. These results indicate that sex differences in this vascular lesion were completely abolished in the ET_B_ receptor-inhibited condition. Therefore, ET_B_ receptors could play an important role in the sex differences observed in the development of balloon injury-induced neointimal formation.

We evaluated the involvement of estrogen-induced vasoprotective effects on this abolition of sex differences. In female wild-type rats, neointimal formation after balloon injury is markedly aggravated by ovariectomy and this aggravation is almost completely reversed by 17*β*-estradiol treatment, clearly indicating that estrogen inhibits neointimal formation after vascular injury in female wild-type rats [8]. Importantly, ovariectomy and 17*β*-estradiol treatment failed to affect the neointimal formation observed in female ET_B_-deficient rats [8]. These findings indicate that estrogen is likely to inhibit neointimal formation after vascular injury via a mechanism that is dependent on ET_B_ receptor-mediated actions since the vasoprotective effects of estrogen after vascular injury were abolished in ET_B_-deficient rats. Furthermore, ET_B_ receptor-mediated actions seem to occur downstream of the vasoprotective effects of estrogen. However, the possibility that marked augmentation of balloon injury-induced neointimal formation by ET_B_ receptor deficiency merely induces functional abolition of the above-mentioned sex differences cannot be ruled out. Further investigations are required to clarify the crosstalk between estrogen receptor- and ET_B_ receptor-mediated actions. We further examined the possible involvement of ET_A_ receptor-mediated actions on the abolition of sex differences of the balloon injury model in ET_B_-deficient rats and found that the aggravation of neointimal formation after balloon injury in female ET_B_-deficient rats was completely suppressed by the blockade of ET_A_ receptors [8]. Thus, we suggest that the augmentation of ET_A_ receptor-mediated actions rather than ET_B_ receptor deficiencies itself contributes to the abolition of the sex differences in ET_B_-deficient rats. 

On the other hand, treatment with a selective ET_A_ receptor antagonist or ET_A_/ET_B_ dual receptor antagonist did not affect neointimal formation after balloon injury in female normal rats, whereas these ET receptor antagonists clearly inhibited neointimal formation in male normal rats [8]. It seems likely that there are sex differences in the vasoprotective effects of ET receptor antagonists and that ET_A_-induced neointimal formation after balloon injury in intact female rats is lower than that observed in male rats.

In clinical fields, several studies showed that postmenopausal women who receive estrogen replacement therapy (ERT) have a substantially lower risk of incidence of cardiovascular disease [51, 52]. However, other clinical trials produced different results. The Heart Estrogen-Progestin Replacement Study (HERS) and Women's Health Initiative (WHI) clinical trial and observational study did not lead to any benefit of ERT [53, 54]. Thus, the effects of ERT on cardiovascular disease are still controversial, and determinations of the mechanisms of estrogen-exhibited vasoprotective effects and the alternative therapy of estrogen in postmenopausal women remain critical issues. Previous studies by us and others suggest that ET_A_ receptor-mediated ET-1 actions may be higher in vascular lesion sites of men and postmenopausal women than those of premenopausal women. Thus, an ET_A_ receptor antagonist may become a useful tool for reducing the risk of cardiovascular diseases after menopause. 

In summary, we demonstrated that ET_B_-receptor actions are involved in the sex differences of vascular injury model. The lack of vasoprotective effects of estrogen and the augmentation of ET_A_ receptor-mediated actions may be responsible for the abolition of these sex differences observed in the ET_B_ receptor-inhibited condition. Although more detailed mechanisms underlying the abolition of sex differences remain to be clarified, we found that modulation of ET-1 and ET receptor expressions by estrogen in injured arteries after vascular injury does not seem to contribute to sex differences in the development of neointimal formation [8]. Finally, efficacious treatment with ET receptor antagonists for vascular diseases may differ between sexes. We expect an accumulation of clinical evidence regarding the relationship between the vasoprotective effects of ET receptor antagonists and sex differences.
